# TGF-*β*1 Induces the Dual Regulation of Hepatic Progenitor Cells with Both Anti- and Proliver Fibrosis

**DOI:** 10.1155/2016/1492694

**Published:** 2015-12-29

**Authors:** Ai-Ting Yang, Dou-Dou Hu, Ping Wang, Min Cong, Tian-Hui Liu, Dong Zhang, Ya-Meng Sun, Wen-Shan Zhao, Ji-Dong Jia, Hong You

**Affiliations:** ^1^Experimental & Translational Research Center, Beijing Friendship Hospital, Capital Medical University, Beijing Clinical Medicine Institute, Beijing Key Laboratory of Translational Medicine in Liver Cirrhosis and National Clinical Research Center of Digestive Diseases, Beijing 100050, China; ^2^Second Department of Gastroenterology, Qingdao Municipal Hospital, Qingdao 266011, China

## Abstract

Transforming growth factor-beta 1 (TGF-*β*1) plays a central role in hepatic progenitor cells- (HPCs-) mediated liver repair and fibrosis. However, different effects of TGF-*β*1 on progenitor cells have not been described. In this study, both *in vitro* (HPCs cocultured with hepatic stellate cells (HSCs) in transwells) and *in vivo* (CCl_4_-injured liver fibrosis rat) systems were used to evaluate the impacts. We found that HPCs pretreated with TGF-*β*1 for 12 hours inhibited the activation of HSCs, while sensitization for 48 hours increased the activation of HSCs. Consistent with these *in vitro* results, the *in vivo* fibrosis rat model showed the same time-dependent dual effect of TGF-*β*1. Regression of liver fibrosis as well as normalization of serum aminotransferase and albumin levels was detected in the rats transplanted with HPCs pretreated with TGF-*β*1 for 12 hours. In contrast, severe liver fibrosis and elevated collagen-1 levels were detected in the rats transplanted with HPCs pretreated with TGF-*β*1 for 48 hours. Furthermore, the TGF-*β*1-pretreated HPCs were shown to deactivate HSCs *via* enhancing SERPINE1 expression. Inhibition of SERPINE1 reversed the deactivation response in a dose-dependent manner.

## 1. Introduction

Hepatic progenitor cells (HPCs) are a group of small epithelial cells that reside in the smallest ducts of the biliary tree in the liver [1]. With advances of research technology, we have come to understand and appreciate the active participation of HPCs in both acute and chronic liver diseases. Depending on the disease process and the concurrent changes in the microenvironment, HPCs are pluripotent under different stimuli and can be classically differentiated into hepatocytes or cholangiocytes, or even myofibroblast cells or cancer cells [2–5].

Upon acute liver injury, when hepatocyte division is severely impaired or blocked, HPCs are activated and proliferate, and the expanding HPCs will begin to infiltrate along the liver plate towards the central vein; consequently, they differentiate themselves into either hepatocytes or cholangiocytes to restore the hepatic parenchyma and liver function [6]. Recent studies have shown the important role of HPCs in injury repair and fibrosis in both experimental models and patients with chronic liver disease [7, 8]. The HPC response correlates with the extent of hepatocellular injury and liver fibrosis [9]. In addition, inhibition of hepatic stellate cells (HSCs) activation or iloprost administration reduces HPCs activation increases of hepatocyte differentiation [10]. HSCs are resident perisinusoidal cells distributed throughout the liver, with a remarkable range of functions in normal and injured liver. During liver injury, various inflammatory and fibrogenic pathways contribute to the activation of HSCs, which increased fibrogenesis and altered matrix degradation; therefore, HSCs can be important therapeutic targets [11].

However, there has been ongoing debate whether the effects of the HPC response in liver fibrosis are antifibrotic or fibrogenic. In the CCl_4_-induced rat model of hepatic failure with a two-thirds hepatectomy, HPCs effectively participated in repairing the damaged liver as shown by our previous study [12]. Our recent data also have shown that transplanted HPCs ameliorate CCl_4_-induced liver cirrhosis. However, a pluripotent differentiation of HPCs was observed. For instance, the expansion of the HPC compartment, which is known as ductular reactions, can clearly lead to transient amplification of the heterogeneous cell population, which is capable of differentiating into liver parenchymal and myofibroblast cells. Ductular reactions are often accompanied with HPCs activation as well as excessive deposition of extracellular matrix (ECM) around the portal areas, suggesting a direct correlation between ductular reactions and periportal fibrosis [5, 13].

Transforming growth factor-beta 1 (TGF-*β*1), a multifunctional cytokine, exerts its biological effects on tissue and organ development, cellular proliferation, differentiation, survival, and apoptosis [14]. In the liver, TGF-*β*1 is hypothesized to serve as the important link among liver regeneration, chronic injury, cirrhosis, and hepatocellular carcinoma. TGF-*β*1 is considered the most potent hepatic profibrogenic cytokine predominantly produced by activated mesenchymal cells upon chronic liver damage [15]. It is reasonable to suggest a regulatory axis from TGF-*β*1 to HPCs and then to HSCs in hepatic fibrosis. Their interrelationship can be dissected via partially mimicking the pathological microenvironment in fibrosis with HPCs exposed to a high concentration of TGF-*β*1.

To investigate the interrelationship among TGF-*β*1, HPCs, and HSCs in the imitated microenvironment, we utilized both* in vitro* and* in vivo* systems. In the* in vitro* study, HPCs pretreated with or without TGF-*β*1 were indirectly cocultured with HSCs. In the* in vivo* study, HPCs pretreated with or without TGF-*β*1 were transplanted into spleen with CCl_4_-induced fibrosis. Furthermore, the mechanisms involved in this regulatory axis from TGF-*β*1 to HPCs and then to HSCs in hepatic fibrosis were studied using an epithelial-to-mesenchymal transition- (EMT-) related polymerase chain reaction (PCR) array.

## 2. Materials and Methods

### 2.1. Coculture of HPCs with HSCs

The rat hepatic progenitor cell line (WB-F344) was obtained from Academy of Military Medical Sciences. The rat hepatic stellate cell line (T6) was kindly provided by Dr. Friedman. Both WB-F344 cells and HSCs-T6 were plated in Dulbecco's modified Eagle's medium (DMEM, Gibco, Grand Island, NY, USA) supplemented with 10% (v/v) fetal bovine serum (FBS, Gibco, Grand Island, NY, USA) (complete medium).

An indirect coculture system was assembled using transwell culture plates (0.4 *μ*m pore size, 6-well Millicell; Millipore, Switzerland). This system allows cells to maintain contact through shared culture medium without mixing the two cell lines; 1 × 10^4^ HSCs were plated on the lower chamber, and 4 × 10^4^ HPCs were plated in the upper insert. The coculture system was maintained in an incubator supplied with 5% CO_2_. For the monoculture groups, 1 × 10^4^ HSCs were cultured in a separate dish and treated with the same medium as for the coculture system.

For the TGF-*β*1 treatment, HPCs were cultured with 10 ng/mL TGF-*β*1 in 5% FBS-DMEM medium for 6, 12, or 48 h. SERPINE1 inhibitor, 1H-indole-3-acetic acid, and *α*-oxo-1-(phenylmethyl)-5-[4-(trifluoromethoxy)phenyl] (PAI-039, Axon, USA, Cell Signaling Technology, Danvers, MA, USA) were added to the medium at a concentration of 10 *μ*M simultaneously with TGF-*β*1 to confirm its blocking and reversing HPC influence on HSCs.

### 2.2. Quantitative Real-Time PCR (qPCR) Analysis of SERPINE1 Expression

Total cellular mRNA was extracted from harvested cells with a Qiagen RNeasy Mini Kit (Qiagen, Hilden, Germany). cDNA was reversely transcribed using the Reverse Transcription System (Promega, Beijing, China). The following primers were used: forward, 5′-TCTCCAGGGGCCCTCTGAGGT-3′, reverse, 5′-TGCCCCTCTCCGCCATCACC-3′ (SERPINE1); forward, 5′-CCTGCCAAGTATGATGACATCAAGA-3′, reverse, 5′-GTAGCCCAGGATGCCCTTTAGT-3′ (GAPDH). SYBR Green-based qPCR was carried out on an instrument (Applied Biosystems, USA) for 2 min at 5°C before incubation for 10 min at 95°C to inactivate the reverse transcriptase, which otherwise interferes with the DNA polymerase. Forty cycles at 95°C for 15 s followed by 60°C for 60 s were performed. The mRNA expression of the target gene was normalized to GAPDH. The relative amounts were expressed as the means ± standard deviation (SD) from three independent experiments.

### 2.3. Western Blotting of Cell Differentiation-Related and Fibrogenic Markers

Cells were washed and lysed with buffer (250 mM NaCl, 50 mM Tris, pH 7.4, 1% Nonidet P40, 5 mM EDTA, 50 mM NaF, and 1 mM Na_3_VO_4_) supplemented with a protease inhibitor mixture (Roche Applied Science, USA). After boiling for 10 min, the lysates were separated in 12% sodium dodecyl sulfate-polyacrylamide gels. The blots were blocked using 5% nonfat dry milk in tris-buffered saline containing Tween 20 for 2 h at room temperature followed by incubation with the primary antibodies against albumin (ALB, diluted 1 : 500, Abcam, Cambridge, UK), *α*-fetoprotein (AFP, diluted 1 : 1000, R&D Systems, USA), *α*-smooth muscle actin (SMA, diluted 1 : 1000, R&D Systems, USA), TIMP-1 (diluted 1 : 1000, R&D Systems, USA), and PCNA (diluted 1 : 1000, Abcam, Cambridge, UK) at 4°C overnight. After washing, the blots were incubated with the appropriate horseradish peroxidase-conjugated secondary antibody (ZSGB Bio, China) for 1 h at room temperature, and reactivity was detected by the Enhanced Chemiluminescence Kit (Thermo Fisher Scientific Inc., USA). After signal detection, the membranes were incubated with an anti-*β*-actin antibody (diluted 1 : 2000, Sigma, US) as a loading control. The western blot detection was repeated three times.

### 2.4. Gene Expression by the EMT PCR Array

Differential expression of EMT genes was analyzed using the Rat EMT PCR Array (PARN-090ZC, Qiagen, Hilden, Germany). RNA was extracted according to standard protocols and converted to first strand cDNA using the RT2 First Strand Kit. The template was added to an instrument-specific, ready-to-use RT2 SYBR Green qPCR Master Mix. The resulting mixture was added to the wells (25 *μ*L/well) of the PCR array plate containing the predispensed gene-specific primer sets (25 *μ*L for the 96-well plates), and PCR was performed. The threshold cycle (Ct) values for all the genes on each PCR array were calculated using the instrument-specific software, and the fold changes in gene expression for pairwise comparison were calculated using the ΔΔCt method.

### 2.5. HPCs Transplantation

All procedures involving laboratory animals were in accordance with guidelines for The Care and Use of Laboratory Animals issued by the Animal Care and Use Committee of the Friendship Hospital, Capital Medical University. Liver fibrosis was induced in male rats by twice-a-week intraperitoneal injections of 0.2 mL/100 g bodyweight of CCl_4_ mixed with olive oil (2 : 3) for 2 weeks. Two days after completion of the CCl_4_ treatment, 5 × 10^6^ HPCs pretreated with TGF-*β*1 for 12 h (TGF-*β*1 pre-HPC (12 h) group, *n* = 6), with TGF-*β*1 for 48 h (TGF-*β*1 pre-HPC (48 h) group, *n* = 6), or without TGF-*β*1 (HPC group, *n* = 6) were diluted in 500 *μ*L of phosphate-buffered saline (PBS) and then transplanted slowly into the rat spleen using a 23-gauge needle [16]. Rats treated with CCl_4_ for 2 weeks and transplanted with 500 *μ*L of PBS were used as the control (PBS group, *n* = 4). Following transplantation, no additional CCl_4_ was administered to any of the four groups. All animals were sacrificed at 4 weeks after cell transplantation, and blood and liver samples were collected upon euthanization.

### 2.6. Examination of Liver Injury

Serum aminotransferase (Jiancheng Institute of Biotechnology, Nanjing, China) levels and albumin (Abcam, Cambridge, UK) were determined as biochemical evidence of liver injury. All blood samples were collected at the end of the experiment.

### 2.7. Histological Examination

Liver samples were fixed in 4% paraformaldehyde, paraffin embedded, and sectioned. Hematoxylin and Eosin and Sirius Red staining were performed. Each sample was independently assessed and scored by two pathologists blinded to the study protocol, according to a fibrosis score system recently published by Cong et al. [17]. The severity of fibrosis was categorized into seven stages (0–6), where 0 indicates no fibrosis and 6 indicates cirrhosis.

### 2.8. Determination of Serum Levels of Collagen-1

Serum levels of collagen-1 were determined using an enzyme-linked immunosorbent assay system (Blue Gene, China) according to the instructions supplied by the manufacturer.

### 2.9. Immunohistochemical Staining of *α*-Smooth Muscle Actin

Immunohistochemical analysis was performed using rabbit anti-*α*-SMA (diluted 1 : 100, Abcam, Cambridge, UK). The detailed method has been published previously [17]. Five fields in each section were randomly selected to calculate the ratio of positive expression area.

### 2.10. Immunofluorescence Staining of SERPINE1

HPCs were incubated in media containing different concentrations of PAI-039 in DMSO for 48 h, fixed with 100% methanol for 5 min at −20°C, and blocked with PBS containing 10% goat serum, 0.3 M glycine, 1% bovine serum albumin, and 0.1% Tween for 2 h at room temperature. The HPCs were then incubated with rabbit anti-SERPINE1 (diluted 1 : 100, Abcam, Cambridge, UK) at 4°C overnight. After washing three times in PBS, the primary antibodies were reacted with the corresponding Alexa Fluor 488-conjugated anti-IgG (diluted 1 : 500, Abcam, Cambridge, UK) at 37°C for 30 min. Sections were examined under an Olympus CX41 fluorescence microscope (Olympus, Japan).

### 2.11. Statistical Analysis

All data are expressed as the mean ± SD from three independent experiments. Differences between mean values of multiple groups were analyzed using the nonparametric analysis of variance test (SPSS Inc., Chicago, IL, USA). Comparison between two groups was made using Student's *t*-test. *P* < 0.05 was considered to be significant.

## 3. Results

### 3.1. TGF-*β*1-Induced HPCs Differentiated into Mesenchymal-Like Cells

**Figure 1:**
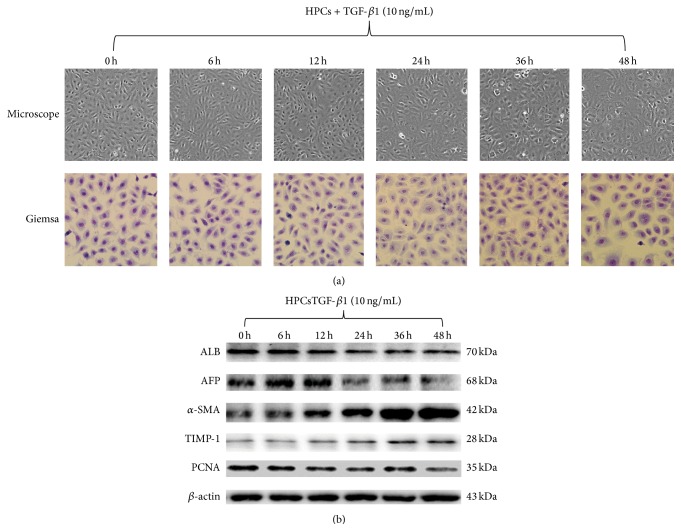
Transforming growth factor-beta 1 (TGF-*β*1) induced a mesenchymal morphology in HPCs. HPCs were cultured in the presence (10 ng/mL) or absence of TGF-*β*1 for 48 h. The phenotypic changes (transition toward a myofibroblast-like phenotype) were evaluated by phase-contrast microscopy and Giemsa staining (a). Changes in differentiation markers were evaluated by western blot (b).

The exposure of HPCs to TGF-*β*1 for 0, 6, 12, 24, 36, and 48 h gradually altered the cell morphology from a typical polygonal shape and cobblestone monolayer appearance to elongated, spindle-shaped cells. As shown in Figure 1(a), we found that HPCs treated with TGF-*β*1 began to present phenotypic changes after 12 h, and remarkable phenotypic changes occurred after 48 h.

Furthermore, we observed the altered expression of differentiation markers in HPCs treated with TGF-*β*1. As shown in Figure 1(b), *α*-SMA was induced as early as 12 h, while the expression of other markers of progenitor cells, including ALB and AFP, was inhibited 24 h after TGF-*β*1 treatment, suggesting that although *α*-SMA was increased at 12 h after TGF-*β*1 induction, the HPCs still possessed the phenotype of stem cells. Since TGF-*β*1 treatment for 12 h induced a high level of *α*-SMA expression compared to treatment for 6 or 48 h, we chose TGF-*β*1 treatment for 12 h for all of the subsequent experiments.

### 3.2. HPCs Pretreated with TGF-*β*1 for 12 h Inhibited HSCs Growth

**Figure 2:**
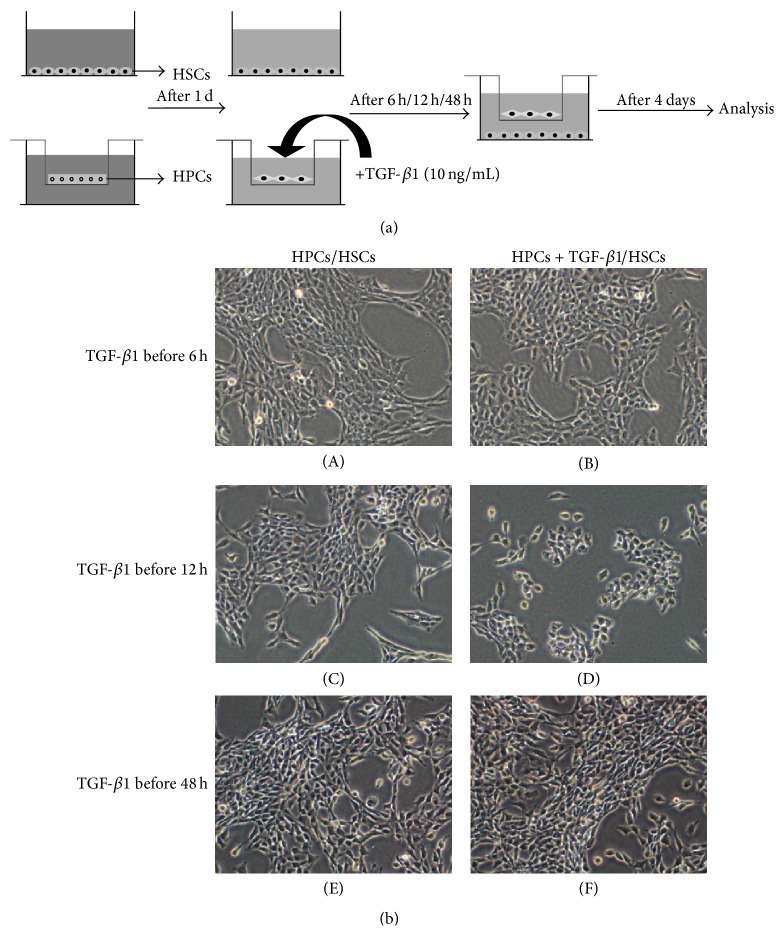
Influence of preexposure of HPCs to TGF-*β*1 for different periods of time on the morphology of HPCs. Schematic of the experimental procedure (a). After exposure to TGF-*β*1 for 12 h, the HPCs were cocultured with HSCs. The HSCs lost their typical spindle shape, while the other groups did not experience any morphological changes (b).

To mimic the* in vivo* microenvironment of fibrosis, we cocultured HSCs and progenitors pretreated with TGF-*β*1 for different periods of time (Figure 2(a)). We observed the effects of HPC exposure to TGF-*β*1 on the activation of HSCs.

The morphological changes of HSCs were only observed after they were cocultured with progenitors pretreated with TGF-*β*1 for 12 h (Figure 2(b)). In this group, the HSCs lost their typical spindle shape and became round or oval with round nuclei. The number of cells did not increase. No morphological changes in the other groups were observed; they still carried a typical HSC appearance, including spindle-shaped, star-shaped, or irregular cell bodies with oval or elongated nuclei. 

### 3.3. Preexposure of HPCs to TGF-*β*1 for Different Periods of Time Caused Different Activations of HSCs

**Figure 3:**
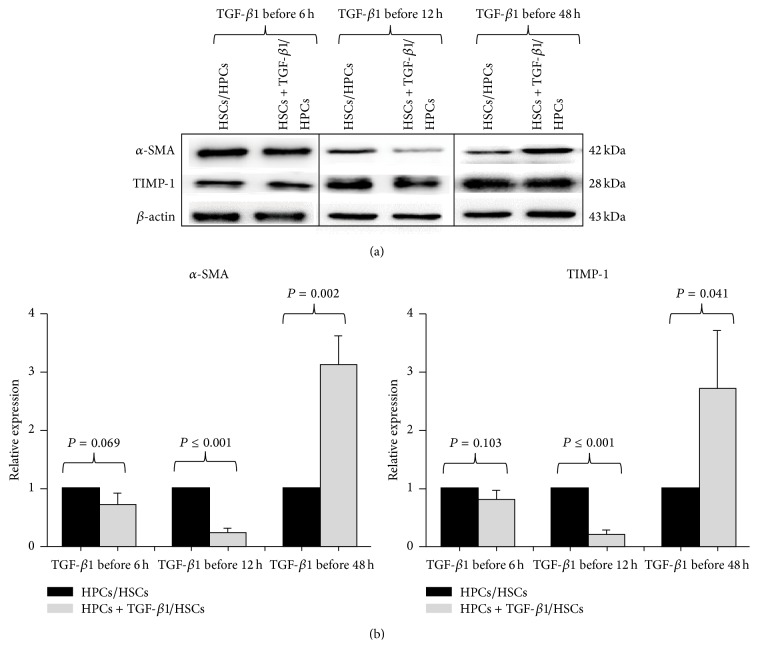
Influence of preexposure of HPCs to TGF-*β*1 for different periods of time on the activation of HPCs. The expression levels of *α*-SMA and TIMP-1 were measured by western blot (a). Quantification of the expression levels after normalization is shown in the lower panel (b). All data are expressed as the mean ± SD from three independent experiments.

Next, we analyzed the expression of fibrogenic genes in HSCs that were cocultured with HPCs pretreated with or without TGF-*β*1 for 6, 12, or 48 h. As shown in Figures 3(a)-3(b), HSCs cocultured with HPCs pretreated with TGF-*β*1 for 6 h showed no significant difference in *α*-SMA or TIMP-1 expression compared with no TGF-*β*1 treatment of HPCs; the HSCs cocultured with pretreated HPCs stimulated with TGF-*β*1 for 12 h had significantly lower *α*-SMA and TIMP-1 expression levels compared with untreated HPCs. However, HPCs pretreated with TGF-*β*1 for longer than 12 h had significantly increased *α*-SMA and TIMP1 expression levels, compared with untreated HPCs. These results demonstrated that preexposure of HPCs to TGF-*β*1 for different periods of time caused differential activation of HSCs.

Animals injected with HPCs exposed to TGF-*β*1 for 12 h had reduced progression of liver fibrosis and improved liver function.

We further evaluated the effects of HPCs exposed to TGF-*β*1 on liver fibrosis by an* in vivo* study. The rat model was injected with the HPCs that were exposed to TGF-*β*1 for 12 or 48 h. These time periods were selected based on the results of the* in vitro* study.

**Figure 4:**
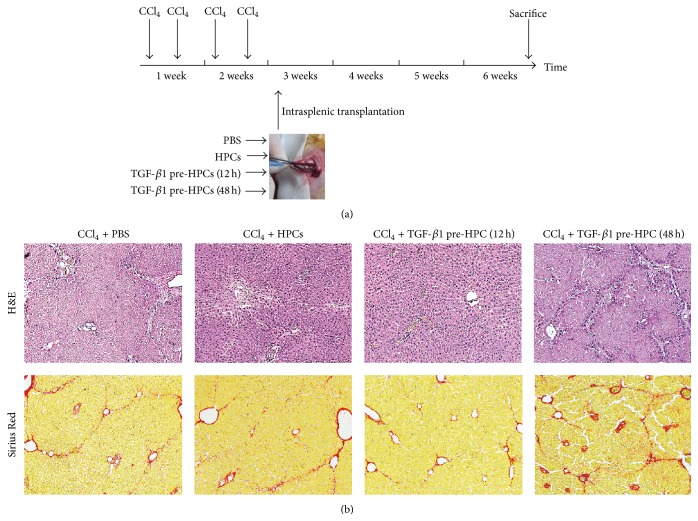
Therapeutic effects of transplanted HPCs, HPCs pretreated with TGF-*β*1 for 12 h, and HPCs pretreated with TGF-*β*1 for 48 h on recovery in the rat model with CCl_4_-induced injury. Schematic description of the experiment (a). Hepatic collagen deposition was determined by H&E and Sirius Red staining (b).

We stained liver sections with H&E and Sirius Red (Figure 4(a)). Semiquantitative grades of liver fibrosis in each group are shown in Table 1. The fibrosis scores demonstrated that the animals with either transplanted untreated HPCs or HPCs pretreated with TGF-*β*1 for 12 h had a reduced amount of fibrosis, compared with the spontaneous regression of fibrosis in the PBS group (*P* < 0.05). Importantly, compared with the HPC transplantation groups, the rats transplanted with HPCs pretreated with TGF-*β*1 for 12 h showed an additional significant reduction in liver fibrosis. The rats transplanted with HPCs pretreated with TGF-*β*1 for 48 h had a significantly increased fibrosis (*P* < 0.05, Table 1).

**Figure 5:**
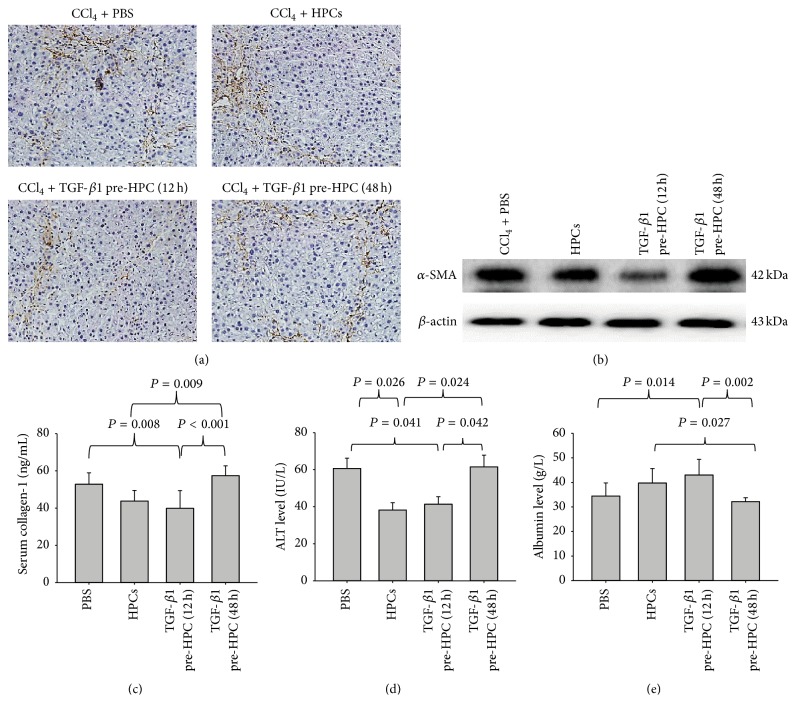
Fibrogenic markers of transplanted HPCs, HPCs pretreated with TGF-*β*1 for 12 h, and HPCs pretreated with TGF-*β*1 for 48 h on recovery in the rat model with CCl_4_-induced injury. The expression of *α*-SMA in liver tissues was detected by immunohistochemistry (a) and western blot (b). The expression levels of collagen-1 in liver tissues were measured by an enzyme-linked immunosorbent assay (c). ALT (d) and ALB (e) in the blood samples collected at the end of the experiment were analyzed. All data are expressed as the mean ± SD from three independent experiments.

Because HSCs are important for liver fibrogenesis, we further examined whether the HPCs had an effect on HSCs activation. The rats transplanted with untreated HPCs or HPCs pretreated with TGF-*β*1 for 12 h showed significant suppression of HSC activation, as indicated by the decreased expression of *α*-SMA and collagen I (Figures 5(a)–5(c)), suggesting that injection of HPCs exposed to TGF-*β*1 for an appropriate time is a novel therapeutic strategy to attenuate liver fibrosis.

In addition, we compared serum ALT and ALB levels across the four experimental groups. The serum ALT levels were significantly lower in the rats transplanted with the HPCs pretreated with TGF-*β*1 for 12 h than in the PBS injection group, and they tended to be even lower in the untreated progenitors-transplanted rats (Figure 5(d)), while the ALT levels in the rats transplanted with the progenitors pretreated with TGF-*β*1 for 48 h were the highest among the four groups. The serum ALB levels were significantly elevated in the untreated progenitors-transplanted rats compared with the control CCl_4_-induced mice. As expected, the ALB level was further elevated in the group transplanted with HPCs pretreated with TGF-*β*1 for 12 h, compared with that in both the PBS- and untreated progenitors-transplanted groups. The rats transplanted with progenitors pretreated with TGF-*β*1 for 48 h showed the lowest levels among the four groups (Figure 5(e)).

### 3.4. SERPINE1 May Mediate the Effects of HPCs Pretreated with TGF-*β*1

**Figure 6:**
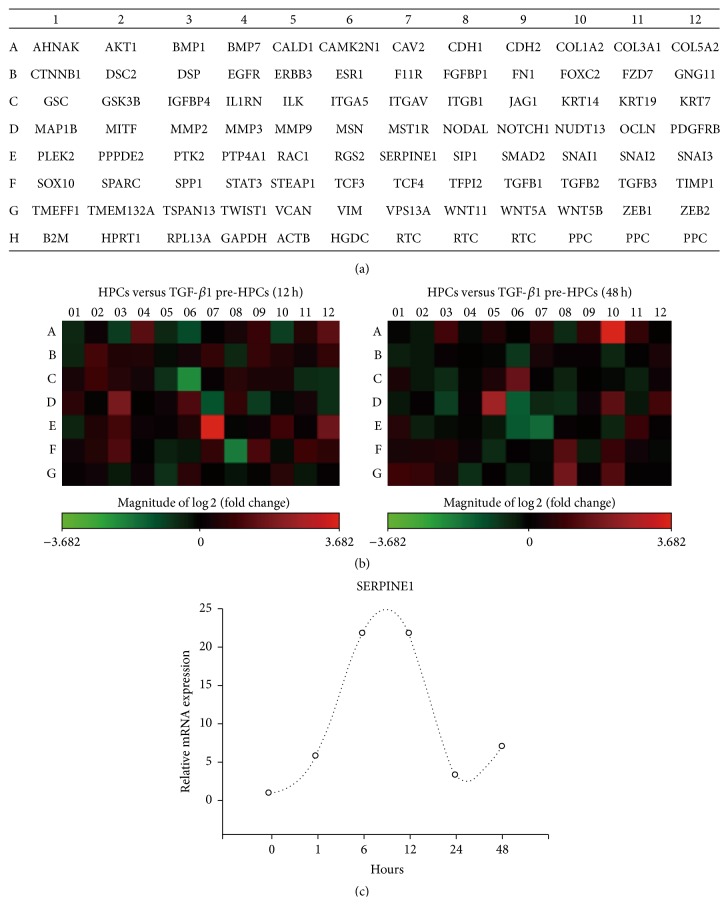
SERPINE1 is required for HPC-mediated amelioration of liver injury induced by an injection of CCl_4_. The layout of the genes included in the EMT Pathway Finder PCR Array (a). The heat map of the variations in the expression levels of 84 genes between control HPCs and HPCs treated with TGF-*β*1 for 12 h or 48 h is shown as a fold increase or decrease (b). Validation of SERPINE1 expression by real-time PCR analysis (c). All data are expressed as the mean ± SD from three independent experiments.

We examined the gene expression profiles using an EMT PCR array and compared the relative expression levels of EMT genes in the HPCs exposed to TGF-*β*1 for 12 and 48 h. The layout of the EMT genes of the PCR array is shown in Figure 6(a). In addition, Figure 6(b) depicts the heat map showing the fold changes in the expression levels between the TGF-*β*1-treated progenitors and the control group. There were many red- and green-colored genes, which signaled both upregulated and downregulated gene expression by the TGF-*β*1-treated progenitors.

The genes with a 5-fold change in the expression level are listed in Table 1. Of 84 EMT-focused genes in this array, the expression of SERPINE1 and ITGA5 was different by 4.5-fold between untreated HPCs and HPCs treated with TGF-*β*1 for 12 h; a 5-fold difference in expression was detected in four genes between HPCs treated with TGF-*β*1 for 12 h and HPCs treated with TGF-*β*1 for 48 h. Collagen-1A2, matrix metalloproteinase-9, and WNT11 were upregulated, and only SERPINE1 appeared to be downregulated in the HPCs treated with TGF-*β*1 for 48 h (Table 2).

Real-time PCR was used to further verify the changed levels of SERPINE1 detected in the PCR array. The expression of SERPINE1 was upregulated in the progenitors treated with TGF-*β*1 for 12 h, and it was downregulated in the HPCs treated with TGF-*β*1 for 48 h, which further confirmed the previous results in the PCR array (Figure 6(c)).

### 3.5. Inhibition of SERPINE1 in HPCs Prevented the Response of HSCs to HPCs

To further confirm the role of SERPINE1 in mediating the response of HSCs to progenitors, we used a chemical inhibitor (PAI-039) that blocks SERPINE1 expression to determine whether the response of HSCs to progenitors could be interrupted.

**Figure 7:**
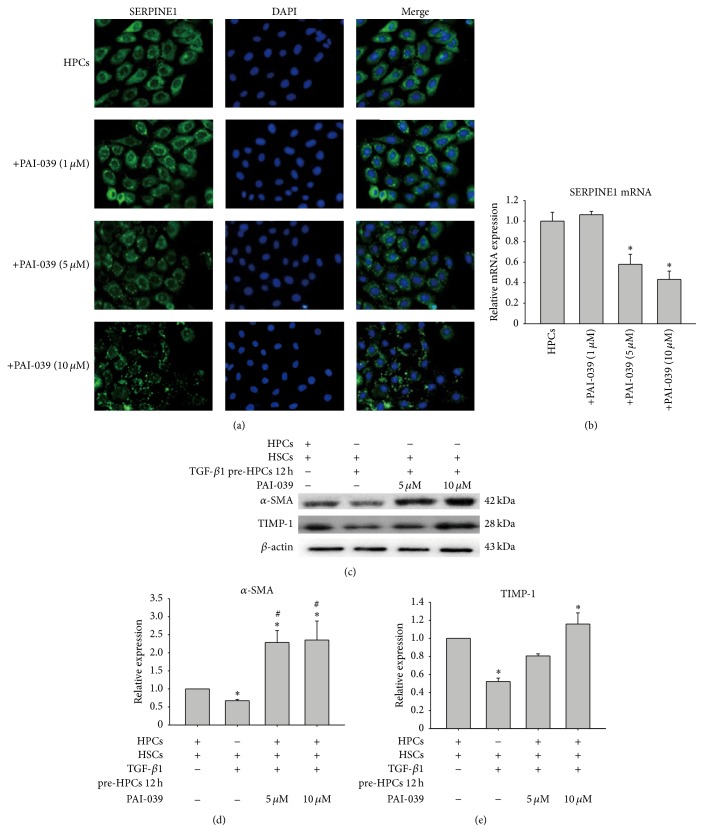
Influence of the SERPINE1 inhibitor on HSC activation after HSCs were cocultured with HPCs pretreated with TGF-*β*1 for 12 h. After incubation with various concentrations of the SERPINE1 inhibitor PAI-039 (0–10 *μ*M) for 48 h, SERPINE1 expression was inhibited significantly as shown by immunofluorescence (a) and real-time PCR (b) analyses. Suppression of SERPINE1 expression prevented the response of HSCs to HPCs. Data are expressed as the mean ± SD from three independent experiments.

We first determined the optimal concentration of SERPINE1 by a cell counting kit-8 assay. Treatment with PAI-039 at ≥15 *μ*M for 24 or 48 h reduced cell viability significantly (data not shown). Thus, we selected 1, 5, and 10 *μ*M PAI-039 to test inhibition of SERPINE1 expression. As shown in Figures 7(a)-7(b), 5 and 10 *μ*M PAI-039 remarkably inhibited SERPINE1 expression in a dose-dependent manner (*P* < 0.05).

Finally, we examined whether inhibition of SERPINE1 affected the response of HSCs to progenitors. As shown in Figures 7(c)–7(e), PAI-039 efficiently blocked the response of HSCs to HPCs treated with TGF-*β*1 for 12 h. These results demonstrated that HPCs inhibited HSC activation largely* via* a SERPINE1-dependent mechanism.

## 4. Discussion

Little is known regarding the effect of HPCs on HSC activation and fibrosis under pathological conditions. TGF-*β*1 is a potent profibrogenic cytokine that is produced by activated mesenchymal cells upon liver injury. We assumed that the increased production of TGF-*β*1 in response to liver injury would regulate the functions of HPCs, thus impacting the activity of HSCs in the repair of the damaged liver parenchyma. We aimed to investigate the potential TGF-*β*1–HPCs–HSCs regulatory axis. For this purpose, we cocultured HSCs with HPCs pretreated with TGF-*β*1 to mimic the pathological conditions with the assembled regulatory axis* in vitro*. In addition, to investigate the impacts of the TGF-*β*1-stimulated HPCs on liver fibrosis* in vivo*, HPCs pretreated with TGF-*β*1 were transplanted into rats with CCl_4_-induced liver fibrosis. Furthermore, the potential role of SERPINE1 in mediating the effects of TGF-*β*1-pretreated HPCs on HSC activation was analyzed. We found that HPCs may function differently, depending on the time that the HPCs were sensitized with TGF-*β*1, and that HPCs may activate HSCs via reducing SERPINE1 expression.

The current study highlighted interesting but complicated interactions between TGF-*β*1, HPCs, and HSCs. It appears that TGF-*β*1 exerted profound impacts on HPCs, and the opposite impacts of HPCs on HSC activation and liver fibrosis were primarily determined by the time of TGF-*β*1 exposure, as shown in both coculture and transplantation studies. Our results demonstrated that a short-term exposure (12 h) of HPCs to TGF-*β*1 led to a reduction of the HSC number with a concomitant decrease in the activation of HSCs; more importantly, it prevented the progression of fibrosis and improved liver function. Meanwhile, a relatively longer exposure (48 h) led to increased activation of HSCs and worsened fibrosis in the liver. These findings suggested that the destination and ultimate function of pluripotent HPCs depend on the exposure to or regulation by TGF-*β*1, and it seems that the exposure time played a defining role in mediating pro- or antifibrogenesis. Our results not only confirmed different impacts on repairing the damaged liver tissue exerted by HPCs but importantly provided the evidence necessary to link the function of HPCs and TGF-*β*1 regulation.

We found that TGF-*β*1 could be a double-edged sword in the fibrotic process. It is known that TGF-*β*1 is a multifunctional cytokine whose function depends on which target cell it binds. It can regulate development, differentiation, regeneration, fibrogenesis, tumorigenesis, and metastasis [18, 19]. What is different from previous findings is that TGF-*β*1 may even exert two opposite regulations of the same HPCs in the progression course of chronic liver disease. TGF-*β*1 is widely regarded as a profibrogenic agent in chronic liver injury, and it stimulates myofibroblasts to produce cytokines and ECM. An overwhelming scar-forming wound-healing reaction can lead to distortion of hepatic architecture [18–20]. But a shorter TGF-*β*1 stimulation of HPCs may preferentially destine the differentiation of HPCs into hepatocytes that can restore the hepatic structure of the repaired tissue. Thus, TGF-*β*1 has beneficial effects. We also considered that TGF-*β*1 signaling proteins play a role in both maintaining the undifferentiated state of cells and initiating differentiation [21]. Nagy et al. have shown that treatment of rat liver epithelial cells with TGF-*β* induced the expression of ALB [22]. Similarly, our previous study demonstrated that connective tissue growth factor (CTGF), a downstream mediator of TGF-*β*1, can induce HPC differentiation into hepatocytes [23]. Further studies found that inhibition of the TGF*β*–CTGF signaling axis by iloprost (an inhibitor of CTGF) resulted in a significant reduction of progenitor cell proliferation [10]. During hepatocyte regeneration and proliferation, TGF-*β*1 has an important tissue-mass-limiting cytostatic effect and controls inflammation by generating regulatory T cells [24]. Considering that TGF-*β*1 may exert antifibrotic activity, a simple inhibition of TGF-*β*1 may not be a wise approach to slow and reverse fibrosis.

**Figure 8:**
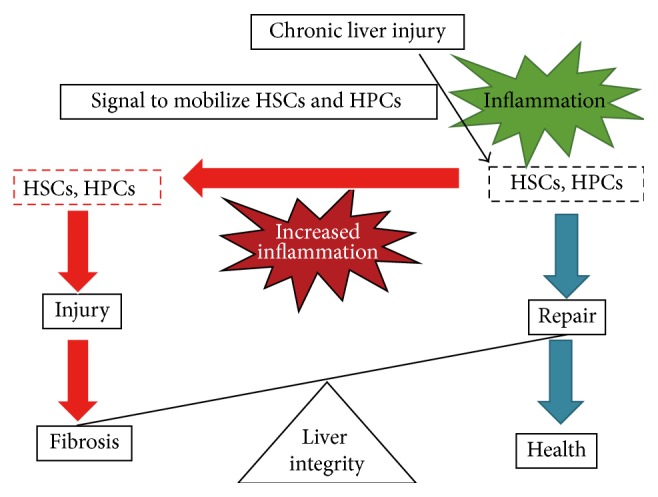
The schematic representation of the balance of HSCs and HPCs between injury and repair.

The surrounding microenvironment is an important determinant to HPC behavior. HPCs are not only involved in tissue repair by differentiation into hepatocytes but also involved in fibrogenesis. As shown in Figure 8, when liver injury occurs, the environment at the injury site is filled with inflammatory chemo/cytokines that are released by infiltrated cells to promote the recruitment of stem or progenitor cells to the site of injury [25, 26]. If a repair is mainly achieved with hepatocytes, the original architecture of the liver will be restored. However, when liver injury is prolonged, the number of progenitor cells is decreased or the cells are not functional so that the repair fails. In this case, the balance is tipped toward a prolonged injury. Cell therapy should be designed to provide the appropriate cells within an adequate time frame and at a sufficient dose to restore the repair potential and capacity [27]. Wu et al. [28] have found that WB-F344 cells exposed to low doses of TGF-*β*1 for 18 weeks acquired tumorigenicity. Taken together, the surrounding microenvironment is the key element to control progenitor cell behavior and the balance between progenitor cell activation, proliferation, and differentiation. Improving the microenvironment should be taken into consideration when progenitor cells are considered as a therapeutic option in chronic liver diseases.

Since preexposure of HPCs to TGF-*β*1 for 12 or 48 h elicited completely different effects on HSC activation, we found that elevated SERPINE1 levels may mediate HPC inhibition of HSC activation. The plasminogen activator and plasmin proteolytic cascades have an important role in stem cell-mediated regeneration, as most regenerative responses are associated with changes in the ECM [29]. von Montfort et al. have found that plasminogen activator inhibitor 1 plays a protective role in CCl_4_-induced hepatic fibrosis in mice [30]. Thus, modulation of SERPINE1 expression may have a therapeutic impact on reversing the fibrotic process.

In summary, differential stimulation of HPCs by TGF-*β*1 for 12 h* versus* 48 h produced opposing anti- and proliver fibrotic effects. Our results further suggest that the antifibrotic function was possibly mediated through the upregulated expression of SERPINE1 in HPCs.

## Figures and Tables

**Table 1:** Quantitative evaluation of liver fibrosis.

Group	Number of rats	Liver fibrosis stage							Average stage
		S0	S1	S2	S3	S4	S5	S6	
PBS	4	0	0	0	1	2	1	0	4.0 ± 0.4
HPCs	6	0	0	3	3	0	0	0	2.7 ± 0.2^*∗*^
HPCs pretreated with TGF-*β*1 for 12 h	6	0	4	2	0	0	0	0	1.7 ± 0.2^*∗*#^
HPCs pretreated with TGF-*β*1 for 48 h	6	0	0	0	0	1	3	2	5.2 ± 0.3^*∗*#$^

The average scores are expressed as mean ± standard deviation (SD) in arbitrary units. *∗* indicates *P* < 0.05, compared to the group of PBS group. # indicates *P* < 0.05, compared to the group of HPCs group. $ indicates *P* < 0.05, compared to the group of HPCs pretreated with TGF-*β*1 for 12 h.

**Table 2:** Variation in the ECM related gene expression between HPCs control and TGF-*β*1-treated HPCs in RT^2^ profiler PCR array.

Group	Position	Gene bank	Symbol	Description	Upregulation or downregulation
HPC + TGF-*β*1 (12 h)/HPC	E7	NM_000602	SERPINE1	Serpin peptidase inhibitor, clade E (nexin, plasminogen activator inhibitor type 1), member 1	12.8
	C6	NM_002205	ITGA5	Integrin, alpha 5 (fibronectin receptor, alpha polypeptide)	−4.7

HPC + TGF-*β*1 (48 h)/HPC	A10	NM_000089	COL1A2	Collagen, type I, alpha 2	40.0
	D5	NM_004994	MMP9	Matrix metallopeptidase 9 (gelatinase B, 92 kDa gelatinase, 92 kDa type IV collagenase)	13.4
	G8	NM_004626	WNT11	Wingless-type MMTV integration site family, member 11	6.0
	E7	NM_000602	SERPINE1	Serpin peptidase inhibitor, clade E (nexin, plasminogen activator inhibitor type 1), member 1	−5.1
